# The role of evidence and context for implementing a multimodal intervention to increase HIV testing

**DOI:** 10.1186/s13012-015-0214-4

**Published:** 2015-02-13

**Authors:** Barbara G Bokhour, Hemen Saifu, Matthew Bidwell Goetz, Gemmae M Fix, Jane Burgess, Michael D Fletcher, Herschel Knapp, Steven M Asch

**Affiliations:** Quality Enhancement Research Initiative for HIV and Hepatitis C, US Department of Veterans Affairs, Bedford, MA USA; Center for Healthcare Organization and Implementation Research, Bedford, MA 01742 USA; Department of Health Policy and Management, Boston University School of Public Health, Boston, MA USA; VA Greater Los Angeles Healthcare System, Los Angeles, CA USA; David Geffen School of Medicine at UCLA, Los Angeles, CA USA; UCLA School of Nursing, Los Angeles, CA USA; VA Palo Alto Health Care System, Menlo Park, CA USA; Division of General Medical Disciplines, Stanford University Medical School, Palo Alto, CA USA

**Keywords:** HIV/AIDS, Health promotion/prevention, Qualitative research, Technology adoption/diffusion, Primary care, Implementation

## Abstract

**Background:**

Increasing the use of routine preventive care such as HIV testing is important, yet implementation of such evidence-based clinical care is complex. The Promoting Action on Research Implementation in Health Services (PARiHS) model for implementation posits that implementation will be most successful when the evidence, context, and facilitation strategies are strong for the clinical practice. We evaluated the relative importance of perceived evidence, context, and facilitation of HIV testing during the implementation of a multimodal intervention in US Department of Veterans Affairs primary care clinics.

**Methods:**

A multimodal intervention including clinical reminders (CRs), academic detailing—providing education sessions for providers—and social marketing to improve HIV testing was implemented in 15 VA primary care clinics in three regions. We conducted qualitative formative and process evaluations using semi-structured interviews with HIV lead clinicians, primary care lead clinicians, nurse managers, and social workers. Interviews were analyzed thematically to identify barriers and facilitators to implementation of HIV testing and how these were addressed by the intervention. Sites were then rated high, medium, or low on the dimensions of perceived evidence and the context for testing. We then assessed the relationship of these ratings to improvements in HIV testing rates found in earlier quantitative analyses.

**Results:**

Sites that showed greatest improvements in HIV testing rates also rated high on evidence and context. Conversely, sites that demonstrated the poorest improvements in testing rates rated low on both dimensions. Perceptions of evidence and several contextual aspects resulted in both barriers and facilitators to implementing testing. Evidence barriers included provider perceptions of evidence for routine testing as irrelevant to their population. Contextual barriers included clinical reminder overload, insufficient resources, onerous consent processes, stigma, provider discomfort, and concerns about linking individuals who test positive to HIV treatment. While most barriers were ameliorated by the intervention, HIV stigma in particular regions and concerns about linkage to care persisted.

**Conclusions:**

Interventions to implement evidence-based practices such as HIV testing can be successful when utilizing proven quality improvement techniques. However, it is critical to address providers’ perceptions of evidence and consider aspects of the local context in order to fully implement new routine clinical practices such as HIV testing.

## Introduction

Primary care providers are asked to attend to a range of evidence-based preventive care tasks as a central aspect of high-quality care. One such preventive care task, routine HIV testing, has received increasing attention since the CDC released a recommendation in 2006 that all patients, age 13–64, receive an HIV test at least once during their lifetime [1]. Correspondingly, in August 2009, the Department of Veterans Affairs (VA) Healthcare System elevated universal HIV testing in primary health-care settings to a top priority. This was in addition to removal of the requirements for written informed consent, pre-test counseling, and post-test counseling for patients who test negative [2].

Changes in policy, however, often do not seamlessly translate into implementation of evidence-based practice, and changing clinical practices is a complex task. Thus, among veterans using VA for their health care, as of 2011 (2 years after the change in policy), only 20% had ever had an HIV test [3].

In the VA, use of electronic clinical reminders (CRs) has become one means to prompt providers to adhere to a wide range of evidence-based practices and improve performance [4]. They are used as reminders and as performance metrics wherein providers are rewarded for meeting particular performance goals.

While careful design of reminders has been shown to be effective [5], research indicates that providers are resistant to these reminders, finding them onerous and interfering with their practice [5,6]. Our prior study to increase HIV testing in two VA primary care clinics found that enhancing implementation of an HIV CR, using a multimodal approach, led to a three- to fivefold increase in the proportion of at-risk patients offered an HIV test [7,8], but did not explicitly address the implementation barriers and facilitators or the interaction between CRs and the other components *of the intervention*.

A subsequent programmatic expansion provided an opportunity to prospectively assess those implementation factors using local and central facilitation strategies across 15 facilities nationwide. We thus undertook a mixed-methods study, quantitatively assessing the relative success of two different implementation strategies in comparison to a no-intervention control and concurrently conducting qualitative formative and process evaluations at each implementation site. The quantitative findings reported elsewhere [9] indicated that while the majority of patients remained untested, after the 6-month intervention, the proportion of patients seen in primary care who had been tested for HIV infection increased by 6.3–9.2% in the two groups of intervention facilities as opposed to 1.1% in the control facilities (*p* < 0.01). Of note, the variation in implementation strategies only partly accounted for the significant variation in the uptake of testing.

In order to more fully understand factors affecting the successful uptake of the implementation, we turned to the qualitative data using the Promoting Action on Research Implementation in Health Services (PARiHS) [10] framework, a conceptual framework for understanding how new evidence-based practices and policies are implemented in clinical settings. PARiHS proposes that implementation success depends on three key features—evidence, context, and facilitation. Evidence refers to provider perceptions of the strength of the evidence for the evidence-based practice being implemented. Context includes aspects of the culture, leadership, and evaluation at the particular site. Facilitation refers to how the evidence-based practice is introduced into clinical practice.

The current paper first describes the qualitative findings in which we utilize the PARiHS framework to understand barriers and facilitators to implementation of HIV testing. Then, consistent with a concurrent intervention mixed-methods design [11], we examine how different contextual factors and perception of evidence relate to the relative success of the implementation efforts described in the quantitative study.

## Methods

We conducted qualitative formative and process evaluations [12] to examine providers’ perceptions of the evidence and context for HIV testing. We then compared these findings to the quantitative findings regarding sites that were relatively more or less successful in increasing HIV testing after implementation of the intervention [9]. The research team was separated into a qualitative Evaluation Team and a Project Leadership Team (PLT—an infectious disease clinician (the grant principal investigator), a nurse researcher, and a social worker) which implemented the intervention. This separation was critical to facilitate participant willingness to candidly discuss their experiences and to minimize bias in the analytic process.

### Setting of research

The intervention was implemented in 2009–2011 in primary care clinics at Department of Veterans Affairs Medical Centers in three regions of the country. These care clinics are staffed by physicians, physician assistants, and nurse practitioners working in teams with nurses and clerk to serve predominantly male, military veterans.

During the time of the study, changes in policies and procedures at national and local facility levels affected the implementation and analytic plans. When the study was initiated, risk-based HIV testing was the standard of care; we targeted our intervention to testing at-risk patients. However, 3 months into the study, VA policies shifted to a recommend routine testing; our implementation project followed suit. The policy change also included a removal of the requirement for written informed consent and pre-test counseling. These policy changes and our subsequent implementation changes are reflected in the qualitative interviews discussed below.

### The intervention

**Figure 1:**
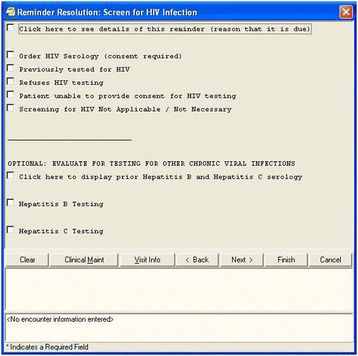
**HIV clinical reminder as seen in the electronic medical record system.**

**Figure 2:**
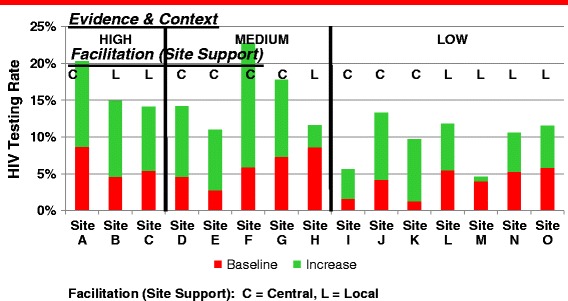
**Evidence**, **Context, and Facilitation and HIV testing rates at 15 sites.**

We implemented a multimodal HIV testing intervention at 15 VA medical centers in three regions. At all sites, a facility-specific study team was established that consisted of an infectious diseases specialist, primary care team leader, and other personnel involved in either the local HIV testing program or primary care clinical operations. The intervention involved 1) implementing a clinical reminder to provide decision support for HIV testing (see Figure 1), 2) a multifaceted provider activation program conducted by the PLT at site visits to each medical center using academic detailing [13-15], 3) social marketing techniques including provision of patient and provider materials, and regular informal discussions about HIV testing to facility and clinic leadership and 4) quarterly feedback reports on rates of testing (see List of intervention components).

List of intervention components:

1. Clinical reminder implementation
  Reminder loaded into electronic medical record system and tested
  Reminder fully activated for all providers on the date of site visit
2. Academic detailing—site visit to conduct in person 1.5-h presentation to primary care staff
  Overview of prevalence
  Benefits of HIV testing
  Success of intervention to date
  Overview of the HIV clinical reminder and how to resolve
  Guidance on informed consent
  Tips for proposing HIV testing to patients
3. Social marketing
  Provider media
    Pocket card—tips for proposing testing; providing HIV education to patients; delivering test results; documenting consent; contact information for local infectious disease clinician
    Informal discussions of the basis for and benefits of increased rates of HIV testing by project staff during frequent ad hoc visits to the primary care clinic and presentations to facility and clinic leadership (central facilitation sites only)
  Patient media
    HIV mini-poster promoting HIV testing

As the overall study goals were to test different methods of implementation, sites were randomized to receive either monthly conference calls and in-person follow-up site visits with the PLT 3–6 months after launch (‘central facilitation’) or only a single conference call with the team 30 days after launch (‘local facilitation’).

Prior to each site visit, the Evaluation Team conducted qualitative interviews with key informants (see below for details) and shared summaries of potential barriers to implementation with the PLT. The Project Leadership Team used this information to tailor their social marketing approach and communication with providers.

### Formative and process evaluation

At each site, the Evaluation Team conducted qualitative formative evaluations at baseline and process evaluations, 4 to 6 months after the site visit and activation of the clinical reminder. The VA central institutional review board approved the study.

### Participant sample and recruitment

We identified a purposive sample of providers at each facility, including the HIV lead provider (the primary HIV clinician at each facility and considered champion of HIV testing), the lead primary care provider, a nurse manager, and where present, a social worker, from each primary care clinic. Providers were recruited via e-mail and phone calls. Verbal informed consent was obtained from all participants. Provider and site names were removed from all data to *ensure* anonymity.

### Data collection

A trained research assistant conducted baseline and follow-up qualitative, semi-structured interviews via telephone in 2009–2011 and lasted 20–25 min. Detailed descriptive notes were recorded at the time of the interview by a second research assistant and reviewed by the interviewer for accuracy [16]. All data presented are taken from *notes* and may not be verbatim quotes. Interview guides (Table 1) were intended to ascertain information about barriers and facilitators to HIV testing.

**Table 1:** **Interview guides for formative and process interviews**

**Phase**	**Questions asked**
Baseline	1. What is your title/job/role?
	a. What role do you play here with regard to HIV disease?
	2. First, we want to know more about how, in general, new clinical tasks are introduced in primary care here. What can you tell me about this?
	3. Now, can you tell me what is going on here in regard to HIV testing?
	a. What are the primary care providers doing with regard to HIV testing?
	i. How likely is a patient to be offered an HIV test in primary care?
	ii. In your experience, how do patients respond when offered a test?
	b. How much of a priority is HIV testing in the context of a clinical appointment?
	4. Is there a clinical reminder here for HIV testing?
	a. If yes:
	i. Have you seen it?
	ii. How do you think this is being received by staff? (How do they feel about it?)
	iii. Who is responsible for resolving the reminder?
	iv. What kind of reminder is in place, routine or at-risk?
	a. If no:
	i. How do you think a CR for HIV testing would be received by staff?
	ii. Who would be responsible for resolving this type of reminder?
	iii. What kind of reminder do you think would be well received—at-risk or routine?
	5. What is the process here for consenting for an HIV test?
	6. What do you think about increasing HIV testing at this facility?
	7. Aside from what we’ve already talked about, what else should we know about your facility when it comes to efforts to increase HIV testing rates (e.g., policies, practices, personnel, patients)?
	8. If you could change 1 policy or practice to increase HIV testing, what would you do?
Follow-up (process)	Today, I would like to discuss with you how the MVQI HIV implementation of this initiative has gone at your facility, and how it has been received by staff
	1. How was this new initiative to increase HIV testing introduced at your facility?
	2. In general, can you tell me about the HIV CR that was implemented at your facility?
	3. What do you think the impact of the CR has been in regards to HIV screening and testing rates here overall?
	4. What did you think about the training session that was conducted by our study staff?
	5. How do providers receive feedback on HIV testing rates?
	6. What do you think the impact of this feedback has been on providers
	7. What problems emerged when trying to increase HIV testing?
	8. In the past four to six months, have you offered HIV tests to any patients?
	9. How do you think the CR changed practice?
	10. What other things have prompted you to offer HIV testing?
	11. What else is going on in regards to HIV testing in the VA or community?
	12. Aside from what we’ve already talked about, what else should we know about your facility when it comes to increasing HIV testing and clinical reminders?

Baseline interview guides included open-ended questions about 1) facility-specific organizational, structural, and attitudinal barriers and facilitators such as the use of a risk-based clinical reminder, ordering of HIV tests, and the consent and counseling processes in place; 2) beliefs about HIV and the importance and prioritization of HIV testing; and 3) what participants thought would facilitate increased HIV testing. We probed for strategies providers used to accommodate to the program needs and tradeoffs, opportunity costs, and work allocation adjustments required to incorporate routine HIV testing into primary care.

*Process evaluation* interviews were designed to understand the effect of the intervention on perceived barriers and facilitators. These interviews addressed changes in beliefs about testing *and* organizational factors for HIV testing and perceptions of the intervention of the CR implementation and the PLT presentation and materials.

### Analysis

The initial analysis was inductive; codes, derived from the data, focused on types of barriers and facilitators described by the participants. This grounded approach was the first analytic step to identify major themes. Subsequently, the PARiHS model and constructs were used as an analytic framework and to organize and interpret these findings.

Inductive analysis involved multiple steps. Two investigators began by assigning descriptive codes to segments of notes. These codes were discussed and reviewed by two additional investigators, and codes were added as subsequent notes were coded. Coded text was reviewed by the Evaluation Team weekly to consolidate codes into discrete themes using constant comparison analysis and tools from grounded theory [17]. Coding focused on the identification of 1) barriers to implementation, 2) facilitating features of the organization that allow for implementation of HIV testing initiative, 3) perceptions of the importance of HIV testing in general and relative to other primary care tasks, 4) unintended consequences of the intervention, and 5) any conflicts that arose during intervention process and how they were resolved.

Second, we turned to the PARiHS constructs to organize and map the findings onto context, perceptions of evidence and facilitation. This step allowed for a further understanding of how barriers and facilitators related to success in testing.

### Assessment of rates of testing improvement

In the quantitative study described above, testing rates were ascertained using administrative data prior to intervention and again 6 months later. Adjusted rates of HIV testing were calculated for each site using logistic regression analyses in which the unit of analysis was the patient who was seen at the VHA facilities in each month, but the HIV Testing Clinical Reminder had not previously been resolved. A full description of these methods and more detailed findings can be found in our previous paper [9].

### Comparison of qualitative and quantitative findings

Using the PARiHS model, we then identified themes that indicated a high vs. a low perception of the evidence and a high vs. a low context for implementation. Two coders, blinded to site performance on testing, independently rated each site based on the extent to which the interviews revealed a high, medium, or low perception of the evidence for testing and high, medium, or low context for testing, where medium indicated a mix of themes endorsed for a dimension. Interrater reliability was high (kappa coefficient 0.89 for evidence, 0.78 for context). Through further discussion, coders came to 100% consensus on the ratings. After each site was rated for evidence and context, sites were unblinded and the context and evidence levels were compared to the changes in testing rates at each site.

## Results

We conducted 50 provider interviews prior to the intervention with 14 HIV lead clinicians, 15 primary care lead clinicians, 15 primary care nurse managers, 3 primary care nurses, and 3 HIV social workers (Table 2). We conducted post-intervention interviews with the same providers. However, as some providers had left the facility or declined participation, only 41 post-intervention interviews were completed. Below, we present the barriers and facilitators we identified in our analysis, grouped according to the PARiHS framework.

**Table 2:** **Distribution of providers by profession and region**

**New England**	**Northeast**	**South Central**
HIV lead clinician, 3	HIV lead clinician, 5	HIV lead clinician, 6
Primary care lead clinician, 2	Primary care lead clinician, 5	Primary care lead clinician, 8
PC nurse manager, 1	PC nurse manager, 5	PC nurse manager, 9
PC nurses, 1	PC nurses, 0	PC nurses, 2
HIV social workers, 1	HIV social workers, 2	HIV social workers, 0

### Barriers and facilitators to HIV testing—qualitative findings

Participants described several barriers to increasing HIV testing including insufficient resources, CR overload, onerous consent processes, stigma and provider discomfort, and concerns about linking individuals who test positive to HIV treatment. They also identified provider education, streamlining the HIV consent and testing process, supportive staff and administration, viewing CRs positively and concern for patient welfare as key facilitators for increasing HIV testing rates. Utilizing the PARiHS framework, we group these barriers below into contextual and evidence categories (see also Table 3). We then discuss how the intervention as the facilitation to HIV testing affected perceptions of these barriers and facilitators.

**Table 3:** **Barriers and facilitators to implementing a multimodal intervention to increase HIV testing grouped by the PARiHS framework**

	**Evidence weak**	**Evidence strong**
Context strong	Little HIV in this population^a^	*HIV testing is critical* ^a^
	Population is elderly and monogamous^a^	*Professionalism of providers—believe that HIV testing is doing the right thing* ^a^
	Testing is a low priority^a^	*Strong clinical champion* ^b^
	Strong clinical champion^b^	*External environment of HIV testing in the community* ^b^
	Lots of current testing^b^	*Organizational support for doing testing* ^b^
	Organizational support for doing testing^b^	*CR viewed as facilitative and not burdensome* ^b^
	CR viewed as facilitative and not burdensome^b^	
Context weak	Little HIV in this population^a^	Agree HIV testing is important^a^
	Population is elderly and monogamous^a^	Evidence considered good for testing^a^
	Testing is a low priority^a^	Difficult to talk about HIV/sex in this region^b^
	Difficult to talk about HIV/sex in this region^b^	Insufficient staff to do testing^b^
	Insufficient staff to do testing^b^	CR overload^b^
	CR overload^b^	Poor organizational structure^b^
	Poor organizational structure^b^	No clinical champion^b^
	No clinical champion^b^	

^a^Indicates elements of evidence; ^b^indicates elements of context. Sites that have strong evidence and strong context (in italics) have the best chance to implement testing.

### Context

#### Contextual barriers

##### Clinical reminder overload

One of the biggest concerns about the intervention was the addition of another clinical reminder to a system perceived as overloaded with reminders for other conditions. Not surprisingly, CR overload was identified as a key barrier prior to HIV CR activation. Participants said:

This will not be received well because there are so many clinical reminders. It will go through the ROOF!

There will be a great deal of unhappiness because there are a lot of primary care providers—CR overload!

In the post-implementation interviews, after the CRs were activated, while three providers reported the HIV CR to be a burden, most staff found it fast, easy to use, and less burdensome than other CRs.

It’s really easy. All you do is click on it and it only takes a minute. It’s not difficult like the other clinical reminders.

It’s actually easier to order an HIV test through the CR rather than without the CR.

##### Linkage to care

Providers expressed reluctance to offer HIV testing to individuals when they felt potentially unable to link positive patients to HIV primary care. This issue existed in sites that were without locally accessible HIV clinics, where the closest HIV specialist was over 100 miles away.

Geography is a big problem. Two-thirds of the patients may not follow up because they do not live in the city, they are noncompliant.

We don’t actually have an HIV clinic…now HIV patients now have to go to [a large southwestern city] for care over 140 miles away.

Thus, providers considered carefully whether to test someone if they felt unable to link them to care. Linkage to care was not addressed in the intervention. Therefore, inaccessibility of HIV primary care services generally characterized as great distances to HIV care facilities remained an important barrier.

##### Informed consent requirements

Some of our interviews were conducted prior to the VA policy change in HIV testing consent requirements that substituted verbal for written consent and removed requirements for formal pre- and post-test counseling. Providers viewed the old consent process as onerous and an important barrier to increasing testing.

Until granted relief for requested [written] informed consent, there will be a struggle at its very best to get more than a handful tested.

Written informed consent is problematic, it’s labor intensive…without it, there will be less of a barrier and HIV testing will be like any other test we do.

Thus, providers indicated that the process of obtaining written informed consent and conducting pre- and post-test counseling was burdensome and suggested that the process be streamlined.

##### Insufficient resources

In general, prior to the intervention, providers indicated that time constraints and lack of resources were key barriers to conducting HIV testing. The providers expressed concern that this addition to their workload would be burdensome and that they did not have sufficient staff to either test or to address the needs of those who would test positive.

We’re starved for time and manpower.

The question is [do] we have enough staff for the influx of patients?

In post-intervention interviews, providers indicated that the streamlined consent and testing processes obviated the need for additional staff, noting that it actually took very little time to satisfy the requirement to offer an HIV test.

##### Stigma/provider discomfort

Providers from Southern sites indicated that HIV stigma was a significant barrier to increasing HIV testing.

There is still discrimination and prejudice in this area.

It seems that HIV testing is something people try to avoid here; providers don’t want to be familiar with it, fear giving back results, dealing with treatment and patients questions regarding HIV.

Stigma was not only about having HIV but also about regional taboos about discussing sexuality. In one particular Southern site, being in the ‘Bible Belt’ meant a general cultural discomfort with discussions of sexuality by both patients and providers. As one provider stated,

We are in the Bible Belt—we’re hesitant to talk about sex, it’s culturally hard and [patients] feel like it’s an invasion of their privacy.

In contrast, Northeastern sites spoke of their more tolerant and accepting culture of HIV screening.

We are pretty are receptive to HIV testing, we’re in [major east coast city].

In post-implementation interviews, there was no indication that concerns about HIV stigma had changed.

#### Contextual facilitators

##### Supportive regional HIV testing culture

A powerful facilitator for HIV testing was an environment that encouraged awareness and screening of HIV. Many interviewees in a large eastern city mentioned that their open-minded, liberal atmosphere allows for HIV discussions, education, and promotion of testing. One provider explained:

[We are] pretty receptive, we’re in [Eastern City]. Patients are aware of HIV disease so it’s positively received. Now more patients are willing to test for it; everyone wants to know their status. Many [Veterans] ask to have the test because of media, people are more educated, and everyone’s talking about it, it’s everywhere.

This city’s significant effort to raise awareness for HIV had helped increase HIV testing and receptivity. Beyond the VA and CDC mandates, regions of this city had an initiative for all adults to be tested for HIV.

### Supportive leadership/staff/administration

Organizational support was frequently mentioned among the key facilitators to increasing HIV testing rates. Generally, providers expressed that supportive staff and administration would facilitate HIV testing: ‘I think senior leadership is very supportive of positive outcomes for patients and very supportive of staff; they’re very engaged and dedicated folks, this initiative will be very successful amongst our staff.’

However, lack of support could yield an ineffective intervention as one provider explained: ‘We don’t have good senior leadership, it [intervention] wasn’t well supported, and therefore it wasn’t well received.’ This particular site had leadership who opposed this project and created ‘so many road blocks’ for the CR to be implemented correctly.

### Streamlining HIV testing and consenting

In interviews conducted after the consent rule changed, providers stated that needing only verbal informed consent made testing substantially easier, and some believed that HIV testing had increased, even prior to the intervention: ‘HIV testing is now streamlined since there is no pre-test counseling and removal of written informed consent. Coupling the removal or written informed consent with this CR has helped to increase HIV testing a lot!’ Although providers were worried about the success of this intervention because of their perceived barriers, most interviewees noted that this streamlined intervention helped to increase their HIV testing.

### Evidence

#### Evidence barriers

##### Older patients are not at risk

When the routine HIV CR was introduced, many physicians opposed HIV testing for older patients and viewed screening them for HIV as ‘unnecessary and laughable.’ As one provider explained:

Older patients are mostly monogamous, so they are low risk, hence low priority. If the patients are younger, sexually active or take drugs then they would be considered high priority.

Perspectives on what age to stop offering HIV tests to patients varied from 50 to 85 years old. Providers often characterized older and younger patients differently in terms of HIV risk.

##### Belief that HIV prevalence is low

At some sites, little priority was placed on HIV testing because providers believed HIV was not prevalent and that patients did not perceive themselves to be at risk. One participant stated:

Increasing HIV testing is not very important… our city doesn’t have a large HIV population.

Another participant noted that providers may not think about HIV testing because at that facility there was no HIV clinic. He explained that doctors screen for diseases that they routinely see:

If an MD walks by a department with a lot of heart attacks, he will screen more for heart attacks. Our HIV patients are taken to a different facility, so we don’t see many HIV cases. Doctors need to overcome their out of sight out of mind obstacle that hinders them from HIV testing.

#### Evidence facilitators

##### Positive attitude towards the HIV clinical reminder

Many providers viewed reminders as a critical way to increase provider awareness of HIV testing and making it a clinical priority, despite the complaints of clinical reminder overload.

[HIV CRs] remind doctors to do what they’re supposed to do.

I think the staff will receive the CR fine, it will be ok; especially since we are having new vets coming in and they’re younger, we have a need for it, it will be positive and helpful.

I would educate staff and recommend all of them to use a CR and give feedback. No doctor is happy about a CR but it is helpful to remind the doctor to do everything they are supposed to do.

In post-implementation interviews, providers reported that the HIV CR increased the likelihood and priority of testing and was helpful by keeping HIV testing in the forefront of their minds when seeing patients. In post-implementation interviews, providers noted that the CR was helpful by keeping HIV testing in the forefront of their minds when seeing patients, despite this absence of a local HIV clinic.

The CR has changed our practice 180 degrees. We weren’t testing before. Unless the providers were being watched, they wouldn’t test.

Providers considered the HIV CR to be an effortless tool that served as a reminder to offer HIV testing without requiring extra time or staff. At some sites, the reminder was initially for at-risk testing and switched to routine testing with the change in the national directive. While both reminders were viewed favorably, the routine reminder was especially favored in the South as routinizing removed the association of testing with risky behaviors, typically taboo topics in the region.

##### Professional ethical responsibility

Concerns for patient welfare and professional responsibility to address patients’ needs were a common theme. Many providers described an obligation to do what was in the patients’ best interest, one stating:

The highest priority for primary care is the welfare of the patient, to do the best job for their wellbeing.

Providers emphasized that the intervention and HIV testing would be supported by staff if the benefit to patients were demonstrated and it was shown to be good medical care.

### Facilitation

The implementation of the CR alongside the social marketing and educational intervention facilitated HIV testing, although some gaps in facilitation remained.

#### Clinical Reminder helps to identify new HIV cases

The CR raised providers’ awareness of the importance of HIV testing. Providers stated that it helped increase the likelihood and priority of HIV testing and identification of new HIV cases. After the intervention, many providers mentioned that they now test everyone at least once, including older patients, because of the routine CR. One doctor explains how the CR caused them to identify an HIV-positive patient that they would consider low risk and previously would never have been tested:

We tested this blind man that you wouldn’t have expected to have HIV, but he was actually HIV positive! We would have never tested him without this CR.

#### Provider education

For providers, HIV-related health education and emphasizing the importance of HIV testing was perceived to be a key facilitator to increasing testing. Providers suggested in pre-intervention interviews that provider and patient education would be critical.

The staff needs to get educated about the significance of routine testing and see [that] education matters, some education materials [are] very compelling.

Education about the reduced burden of testing when written consent requirements were lifted was also considered critical.

There needs to be a shift in the providers’ mindset because historically HIV testing has been seen as time intensive; if providers see only 1 side that its routine without understanding that it’s now less burdensome then providers will still hesitate to test, so doctors need to realize that it’s not as labor intensive and apply it to their routine care.

While overall education about the value of testing was important, other providers noted that further training was necessary to handle giving results.

I think for giving the test results, there needs to be a technique of how to give positive results; we need to have training on how to give positive results to patients.

Post-intervention, providers viewed the CR coupled with the educational social marketing approach as the most influential facilitators of the intervention. The presentations were seen as stimulating awareness of undetected HIV cases and promoting the availability of antiretroviral drugs at the VA which are critical for veterans who are HIV positive.

#### Relationship of evidence, context, and facilitation to HIV testing rate improvements

All sites demonstrated improvement in HIV testing rates. Figure 2 shows the relative rates of improvement and ratings on evidence and context. Sites that showed greatest improvements in HIV testing rates also rated high on evidence and context. Conversely, sites that demonstrated the poorest improvements in testing rates rated low on both dimensions.

We did observe some heterogeneity in that performance at some sites was not easily explained based on these dimensions, indicating that other factors not accounted for in the qualitative design influenced the testing rates for that facility.

Most notable is the site that demonstrated the greatest improvement was rated medium for both evidence and context. It is important to note that this was a central facilitation site; thus, ongoing facilitation by the Project Leadership Team may have had important influence in improving testing rates. Two sites which were rated low on both evidence and contextdemonstrated moderate improvements in testing rates; however, it is notable that the baseline testing rates at these sites were low, leaving greater room for improvement. Both of these sites also were central facilitation sites, indicating the potential effect of ongoing support for testing from the central Project Leadership Team. Notably, in our interviews, participants did not discuss the follow-up from the Project Leadership Team when discussing initiatives.

## Discussion

Testing for HIV infection is one of many tasks that primary care providers are asked to incorporate into their already busy clinical encounters. In our study, the PARiHS model provided an excellent framework for understanding how different barriers and facilitators to testing impacted the effectiveness of implementation. Providers described key issues surrounding relevance of evidence for HIV testing in their population, contextual barriers to testing including staffing, clinical reminder overload, and key facilitators to achieving increased testing rates at their facility. These perceptions were related to the success of the intervention as measured by improvements in HIV testing rates. In follow-up interviews, we found that many of the barriers were successfully addressed using the social marketing, provider education, and clinical reminder systems that were put into place, while a few barriers remained pertinent. Moreover, ongoing support from the centralized project team further facilitated increased testing, even in sites where perceptions of evidence and context were not high.

Providers found the facilitation provided through education in the site visit presentations valuable, providing salient evidence for both at-risk and routine HIV testing. As providers began offering routine testing, identification of HIV-positive patients who were not considered to be at risk further solidified their confidence in the evidence for testing. The use of these types of exemplars of the value of routine testing may provide further evidence to providers at low prevalence sites.

Contextual barriers such as the perception of the CR as burdensome were ameliorated by the design of the reminder which providers found user-friendly. As electronic health record clinical reminders spread, providers are both intrigued by their potential to aid care and aggravated by their insensitivity to context and clinical priorities [18,19]. In this qualitative study, we confirmed previous findings that providers were initially reluctant to adopt additional electronic efforts to manage their care, but that, when ensconced in a multimodal effort to improve HIV testing, their attitudes were more complex. The change in requirements for consent was consistently viewed as a significant facilitator to testing. Thus, our findings support efforts to reduce the documentation burden sometimes required for routine tests.

Nonetheless, our intervention was not successful in ameliorating all contextual factors. In particular, concerns surrounding discussing HIV testing due to persistent stigma remained. In more conservative regions, providers described discomfort in having conversations about sexuality, resulting in reluctance to offer an HIV test. Burke et al. found that across practice settings, fear or concern about offending patients was a significant barrier to testing [20], and regional cultural differences may intensify this barrier. Efforts to change this cultural barrier may require more intensive and culturally sensitive interventions, beyond those in our evidence-based presentations. Reducing stigma associated with HIV in general may reduce provider resistance to offering HIV testing and increase acceptability among patients. The VA recommendation for routine offers of HIV testing to all veterans, independent of risk factors, has been identified as a means of destigmatizing HIV testing. Yet, this may be insufficient in a culture in which having HIV remains highly stigmatized. Several providers indicated that increasing patient knowledge through education about HIV testing would make testing more routine and may help destigmatize HIV testing. However, just as with provider activation, patient activation must consider cultural variations and sensitivities.

Another contextual barrier that persisted was concern regarding linkage to care. Particularly in sites where patients would need to travel long distances to receive care, providers were concerned that they would not be able to successfully link patients who tested positive to appropriate HIV care. Putting into place clear mechanisms for getting patients into HIV care may facilitate increased testing. Thus, future implementation would need to include providing guidance to providers regarding how to link patients in unique facilities to HIV specialists and treatment.

Having a local clinical champion and opinion leaders advocating for increased testing as well as support of the leadership was also critical to successful implementation. Consistent with diffusion of innovation theory, such opinion leaders positively influenced the adoption of a given intervention by other members of the organization [21,22]. Ongoing support from a more centralized team may also further increase success as noted even in sites where perceptions of evidence and context were not high.

Other studies have found similar barriers to provider engagement with HIV testing including concerns about insufficient time, lack of knowledge or training, and onerous consent processes [20,23]. Our sites varied substantially in the types of barriers faced by clinicians. The customization of program implementation based on the rapid analysis of the formative interviews is likely to have further enhanced the effectiveness of the intervention. Thus, we utilized this data in the *process* of implementation, not solely as a way to assess how barriers and facilitators contribute the endpoint of the intervention. We found that the use of this tailored social marketing and academic detailing was successful in shifting provider perceptions of evidence for testing and accommodating to the context.

### Limitations

There are some limitations to our study. It is not clear if these findings are easily generalizable to settings outside the VA, which has some distinct advantages for this type of intervention. The VA is unique in its comprehensive medical record system with established clinical reminders and the readily available comprehensive care for HIV including low-cost medications. Other settings might also have additional barriers in cost and payment for testing and treatment, issues that are minimized in the VA. Our interviews were limited to three regions of the country; other issues could arise in other regions. Additionally, we only interviewed three providers at each site; interviewing more primary care front line providers may have given us different insights into their clinical practices. Also, the relationship between evidence, context, and increases in testing is modest and does not fully explain the observed heterogeneity of testing increases. Finally, our conclusions regarding how the qualitative findings informed the intervention are limited in that we did not collect data to evaluate this aspect of the study; such data would have added to our understanding of the effect of the intervention. Future studies of implementation should include rigorous observation of implementation to ascertain how implementation teams can optimally utilize qualitative findings in implementation.

## Conclusion

Implementation of important evidence-based practices such as HIV testing is a complex endeavor. Providers’ perceptions of the evidence base and how context supports or hinders implementation are critical to the success of any new initiatives. The PARiHS framework to assess barriers and facilitators is useful for understanding how different facilitation strategies may be effective in implementing disease screening and testing in primary care. While this study focused on HIV testing, similar barriers and facilitators may emerge with new testing initiatives such as recent efforts to increase screening for hepatitis C in certain populations.

The use of social marketing and academic detailing has great potential to improve preventive care practices such as HIV testing. Providers indicated that the multimodal intervention was effective in addressing many pre-implementation *perceptions about evidence and the burden of an additional clinical reminder*. Future intervention, however, must address *contextual issues* such as stigma and communication about sexuality in regionally reserved settings and barriers to connecting HIV-positive patients to services. Our findings demonstrate that interventions to increase HIV testing can be successful when utilizing proven quality improvement techniques. By addressing providers’ perceptions of the evidence and taking into consideration the local context, care systems may be better situated to decide where to provide additional resources to support changes in practice.
